# *In Utero* Electroporated Neurons for Medium-Throughput Screening of Compounds Regulating Neuron Morphology

**DOI:** 10.1523/ENEURO.0160-23.2023

**Published:** 2023-08-28

**Authors:** Aidan M. Sokolov, Mariana Aurich, Angélique Bordey

**Affiliations:** Departments of Neurosurgery, and Cellular and Molecular Physiology, Wu Tsai Institute, Yale University School of Medicine, New Haven, CT 06520-8082

**Keywords:** CellProfiler, dendritogenesis, drug, hypertrophy, mTOR, neurodevelopment

## Abstract

Several neurodevelopmental disorders are associated with increased mTOR activity that results in pathogenic neuronal dysmorphogenesis (i.e., soma and dendrite overgrowth), leading to circuit alterations associated with epilepsy and neurologic disabilities. Although an mTOR analog is approved for the treatment of epilepsy in one of these disorders, it has limited efficacy and is associated with a wide range of side effects. There is a need to develop novel agents for the treatment of mTOR-pathway related disorders. Here, we developed a medium-throughput phenotypic assay to test drug efficacy on neurite morphogenesis of mouse neurons in a hyperactive mTOR condition. Our assay involved *in utero* electroporation (IUE) of a selective population of cortical pyramidal neurons with a plasmid encoding the constitutively active mTOR activator, Rheb, and tdTomato. Labeled neurons from the somatosensory cortex (SSC) were cultured onto 96-well plates and fixed at various days *in vitro* or following Torin 1 treatment. Automated systems were used for image acquisition and neuron morphologic measurements. We validated our automated approach using traditional manual methods of neuron morphologic assessment. Both automated and manual analyses showed increased neurite length and complexity over time, and decreased neurite overgrowth and soma size with Torin 1. These data validate the accuracy of our automated approach that takes hours compared with weeks when using traditional manual methods. Taken together, this assay can be scaled to screen 32 compounds simultaneously in two weeks, highlighting its robustness and efficiency for medium-throughput screening of candidate therapeutics on a defined population of wild-type or diseased neurons.

## Significance Statement

Preclinical studies and screens often rely on cell lines and traditional techniques that are time consuming and introduce human bias during analysis compared with automated methods. Some of these techniques include the manual tracing of cells to quantify morphologic changes in response to various treatments. We developed an assay that allows medium-throughput morphologic analysis in a selective population of neurons *in vitro* while expediting data collection and analysis through automation. This assay is modifiable and is applicable for a wide range of disease conditions.

## Introduction

Several neurologic disorders are associated with abnormalities of neuronal development. One such group of neurodevelopmental disorders are mTORopathies that arise from gene variants leading to increased mTOR activity and epilepsy (Crino et al., 2002). mTOR hyperactivity occurs in neocortical pyramidal neurons and results in increased neuronal soma size, dendritic complexity, and alterations in connectivity, all of which contribute to epilepsy (Feliciano et al., 2013). Although one mTOR blocker, everolimus, is approved for the treatment of epilepsy in one of these disorders, tuberous sclerosis complex (Franz et al., 2021), this drug has severe side effects and seizures remain in most patients. While research has focused on developing novel drugs and treatment strategies, screening their efficacy *in vivo* has remained challenging considering that monitoring seizure activity is difficult and labor intensive. An alternative is to use a medium-throughput to high-throughput phenotypic assay to assess the effect of novel drugs or knock-down of specific molecules on the morphologic development of neurons expressing hyperactive mTOR.

The pharmaceutical industry as well as academic research laboratories have developed phenotypic assays to efficiently analyze the effect of many compounds on cells. These assays are critically important to investigate novel treatments in a wide range of disorders. For neurite outgrowth-based assays, various neuronal systems have been implemented. This includes the use of neuronal cell lines, such as SH-SY5Y cells, which have been differentiated into neurons and immunostained for neurite markers (Dravid et al., 2021; Schikora et al., 2021). Others have used nucleofected cortical neurons in 96-well plates (Blackmore et al., 2010), sparsely transfected primary neurons using automated imaging and analysis (Sharma et al., 2012), and human iPSC-derived neurons (primarily interneurons, with some Layer V cortical pyramidal-like neurons) immunostained for neurite markers (Sirenko et al., 2014; Sherman and Bang, 2018). In addition to using high-throughput automated imaging, studies have incorporated autonomous computational assays for quantitative analysis (Wu et al., 2010; Sharma et al., 2012; Schikora et al., 2021). Although some studies have achieved sparse labeling of neurons, which improves image analysis, none have labeled specific neuronal populations.

Genetic manipulation via *in utero* electroporation (IUE) offers a reliable method to selectively label distinct populations of cells. IUE is a technique involving the injection of DNA plasmid into the ventricles of embryos and applying an electrical current that creates transient pores in the cell membrane. This allows the DNA to enter the targeted neural progenitors lining the ventricle. For example, IUE at embryonic day (E)15 will specifically label Layer II/III cortical pyramidal neurons (Molyneaux et al., 2007).

Here, we provide proof of principle for the use of specific sequential strategies using IUE to induce a disease phenotype in a subset of murine cortical neurons, with the purpose of testing numerous compounds on diseased neurons in a medium-throughput manner using an IN Cell Analyzer 2200 Imaging System and a customized CellProfiler pipeline (Stirling et al., 2021).

## Materials and Methods

### Animals

Experiments were performed according to guidelines set forth by the Yale University Institutional Animal Care and Use Committee and National Institutes of Health *Guide for the Care and Use of Laboratory Animals*. E14 pregnant CD-1 mice were obtained from Charles River Laboratories. Mice were housed in littermate groups under pathogen-free conditions with a 12/12 h light/dark cycle.

### *In Utero* electroporation

IUE was performed by aseptic survival surgery on a total of three E15 pregnant CD1 mice, 1 d postacclimation to their cage. Weight and approximate age were documented before surgery. Mice were injected with 10 mg/kg buprenorphine 30 min before anesthetization. Mice were placed into an anesthesia induction chamber and 3% isoflurane was applied. Following appropriate anesthesia, mice were placed onto a predisinfected surgical table with a heat source and attached to a nose cone with 2–3% isoflurane flow. Mouse incision regions were shaved, and the skin prepared for aseptic surgery. A midline ventral laparotomy was performed. Uterine horns were slowly removed with ringed forceps. DNA solution (∼1–1.5 μg/μl) containing a dual expression plasmid encoding a constitutively active Rheb, mutant Y35L with tdTomato (T2A) under the chicken β-actin promoter with CMV enhancer (CAG) promoter diluted in PBS with 0.1% fast green was prepared before surgery. DNA solution (1.5 μl) was injected into the lateral ventricle using a sterile pulled glass pipette. Electrodes (model 520; BTX) were soaked in 0.9% saline solution and placed on the heads of the embryos, and five, 40-V square pulses of 50-ms duration with 950-ms intervals were applied using a pulse generator (ECM830; BTX). After injection and electroporation, the incision was closed with absorbable sutures and skin closed with 9-mm autoclips. Mice were monitored for the following 2 d. The date of pup birth was recorded, and mice killed at the indicated time point neonatally.

### Microdissection and cell culture

Hibernate E (ThermoFisher Scientific; A1247601) with 2% B27 (ThermoFisher Scientific; 17 504–044) and 2 mm GlutaMAX (ThermoFisher Scientific; 35 050–061) was first placed on ice, and papain digestion solution (Worthington; LK003176) was warmed to 37°C for 30 min before use. Mice were screened following IUE, and at postnatal day (P)1–P2 the brains of IUE-positive mice were collected and placed in a 60 mm-diameter Petri dish with cold Hibernate E solution. The positive somatosensory cortex (SSC) region of three to four brains were microdissected and cortices placed in 5 ml of cold Hibernate E solution on ice in a 15-ml conical tube. Dissected cortices were kept in Hibernate E solution on ice for the duration of all microdissections. Next, the Hibernate E solution was carefully removed, and 5 ml of prewarmed and activated papain digestion solution was added to enzymatically digest the cortices. The cap of the tube was sealed with Parafilm, and then incubated in a 37°C water bath for 15 min. At this step MEM solution (ThermoFisher Scientific; 11095-080) with 0.6% wt/vol of D-Glucose (Millipore Sigma; G8769) and 5% fetal bovine serum (ThermoFisher Scientific; 10082147) was prewarmed. After 15 min, the tube was removed from the water bath, sprayed with 70% ethanol, and the papain digestion solution was removed. A total of 10 ml of prewarmed Hibernate E solution was added. Once the cortices settled to the bottom of the tube, this process was repeated to wash out the remaining papain digestion solution. Finally, the supernatant was removed and replaced with 5 ml of prewarmed MEM solution. Cortices were gently triturated four times using a pipette bulb with a large bore sterile fire-polished glass Pasteur pipette (∼1 mm in diameter) and this was repeated with a slightly smaller bore sterile fire-polished glass Pasteur pipette. The tubes were then centrifuged at 400 × *g* for 2 min, the supernatant removed, and 3–8 ml of MEM solution added (volume depending on size of tissue microdissected and the number of brains used) followed by gentle trituration to resuspend cells. The MEM solution containing suspended cells was then transferred into a new 15-ml tube though a 40-μm cell strainer (ThermoFisher Scientific; 22363547).

The cells were counted using a hemocytometer and cultured in a glass bottom 96-well plate (Cellvis; P96-1.5H-N) pretreated with 50 μg/ml Poly-D-Lysine (ThermoFisher Scientific; A389890-01) and 30 μg/ml laminin (Millipore Sigma; 11243217001) at a density of 30 000 cells per well. Two hours later, half the media was replaced with neurobasal media (ThermoFisher Scientific; 21103-049) containing 2 mm GlutaMAX and 2% B27. For neuron maintenance, half of the media was replaced with an equal amount of fresh neurobasal solution daily. Cells were fixed with 4% paraformaldehyde (PFA) for 30 min at the conclusion of the experiment.

### Immunocytochemistry

Cells were treated in blocking solution composed of 1× TBS (ThermoFisher Scientific; J60764) with 0.1% Tween 20 (Millipore Sigma; P7949) and 2% BSA (Millipore Sigma; A7906) for 1 h and incubated overnight in the primary antibody against RFP (1:500; Rockland; 600-401-379). Cells were again washed three times for 10 min each in wash buffer composed of 1× TBS with Tween 20, and again incubated overnight in anti-rabbit 555 secondary antibody (1:500; ThermoFisher Scientific; A32732).

### Drug treatment

A total of 100 nm Torin 1 (Millipore Sigma; 475991) or an equimolar amount of DMSO (0.01%, controls) was added to neurons 2 h after seeding and replaced daily along with half the media. Drug treatments were done in four wells per condition. Cells were collected at 3 d *in vitro* (DIV) for drug treatments.

### Automated imaging

**Figure 1.**
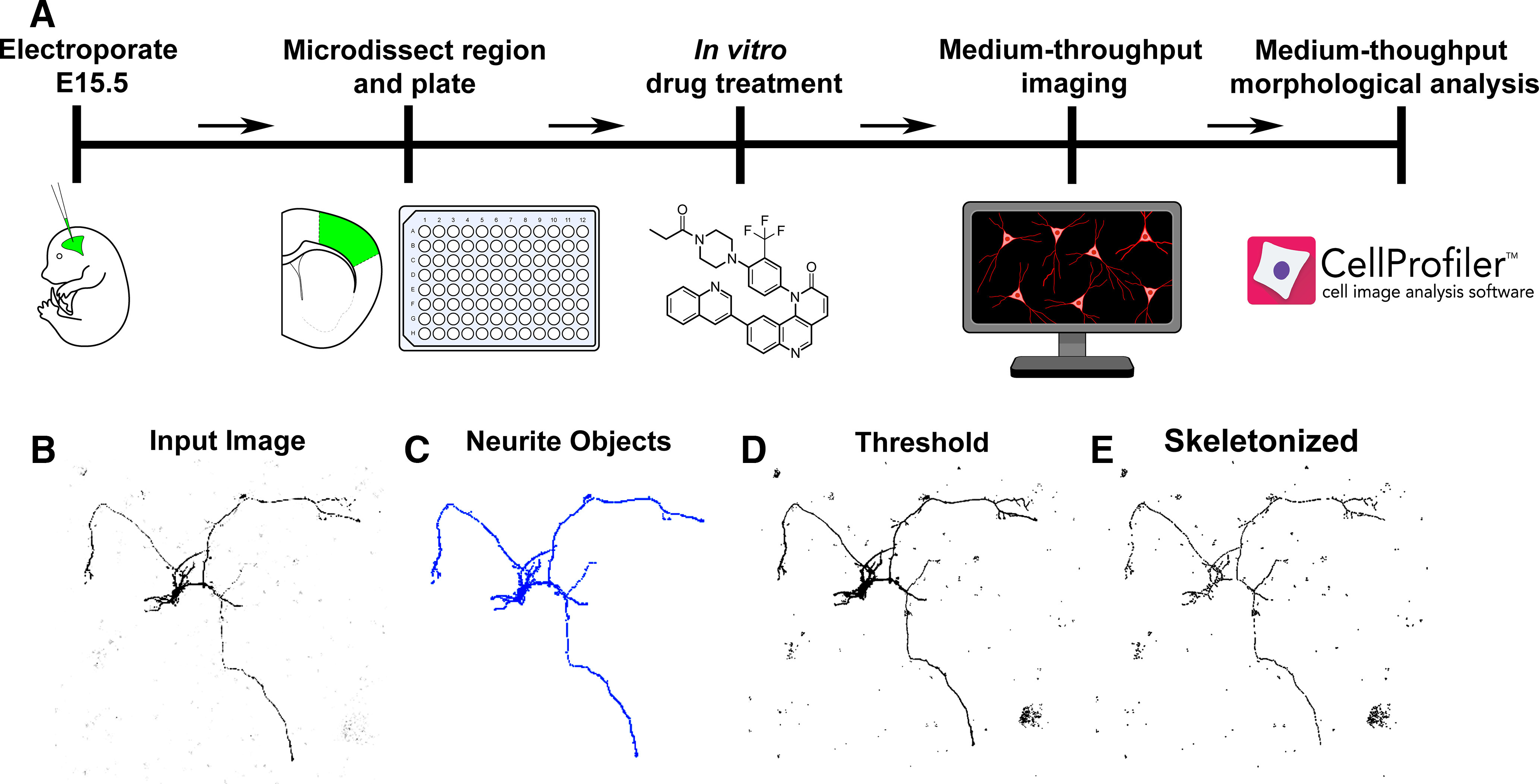
Diagram of the experimental paradigm. ***A***, Summary of proposed steps for our medium-throughput phenotypic assay on primary neurons. ***B–E***, Representative image (***B***) undergoing postprocessing in CellProfiler (***C–E***) for automated measurement of morphologic properties.

Using a General Electric IN Cell Analyzer 2200 Imaging System, images were acquired in an automated fashion (Fig. 1). Fluorescent (CY3) images were acquired at a 10× magnification with a 450-ms exposure time in a grid like pattern for each well to avoid overlap.

### Automated neurite and soma measurements

For automated neurite measurements, a custom pipeline in CellProfiler was developed to measure and export raw data containing both the total neurite length per cell and the number of terminal branches per cell. The settings are as follows: neurites suppressed for identification of soma as primary object (diameter, 12–45 pixels), neurites re-enhanced, and neurites identified as secondary objects based on primary object (soma) location. To convert the image to binary and skeletonize the neurites, we used an adaptive threshold strategy with a smoothing scale of 0.5, a correction factor of 2, a lower and upper bound on threshold of 0.05–1.0, and 10 pixels as the adaptive window size. Finally, the skeletonized neurites were automatically measured using the “measure object skeleton” and “measure object size shape” modules, and readouts such as total neurite length per cell and the number of terminal branches were exported to excel for statistical analysis by the “export to spreadsheet” module.

For automated soma size measurements, another CellProfiler pipeline was generated, where neurites were suppressed using the enhance or suppress features module, and primary objects (soma) were identified. Identification parameters include a 10- to 50-pixel diameter, an adaptive minimum cross entropy threshold with a smoothing scale of 2, a threshold correction factor of 1, a lower and upper bound on threshold of 0.35–1.0, and the shape method to distinguish clumped objects. Finally, the “measure object size shape” module was used to automatically measure the pixel area of primary objects (soma) and the results were exported to excel for statistical analysis. Pipeline files can be found on the CellProfiler website for download.

**Figure 2.**
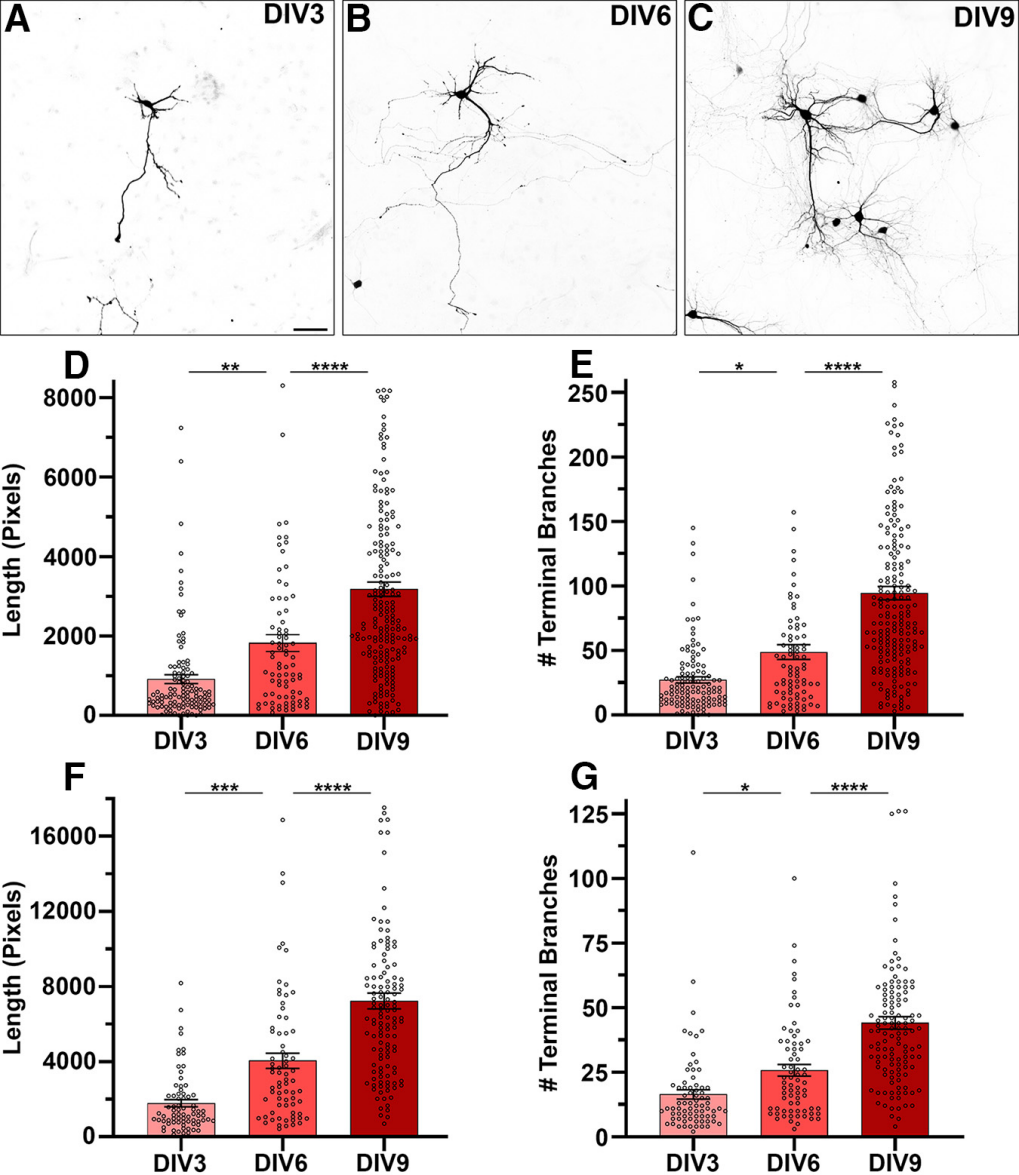
Rheb^Y35L^ neuron neurite outgrowth over time. ***A–C***, Rheb^Y35L^ primary mouse cortical neurons after 3 (***A***), 6 (***B***) and 9 (***C***) DIV. ***D***, ***E***, Automated measurement of total neurite length per cell (***D***) and terminal branch number per cell (***E***) using CellProfiler (DIV3: *n* = 116, DIV6: *n* = 81, DIV9: *n* = 198). ***F***, ***G***, Manual measurement of total length per cell (***F***) and terminal branch number per cell (***G***) using FIJI (DIV3: *n* = 74, DIV6: *n* = 72, DIV9: *n* = 126). One to eight individual points from DIV6 and DIV9 conditions were omitted from the graphs by *y*-axis truncation to better visualize the mean (bar) and the change between conditions. Scale bar = 50 μm. 1 μm = 1.5384 pixels. DIV = days *in vitro*. *p < 0.05, **p < 0.01, ***p < 0.001, ****p < 0.0001. Significant differences were determined by one-way ANOVA with Tukey’s *post hoc* test.

**Figure 3.**
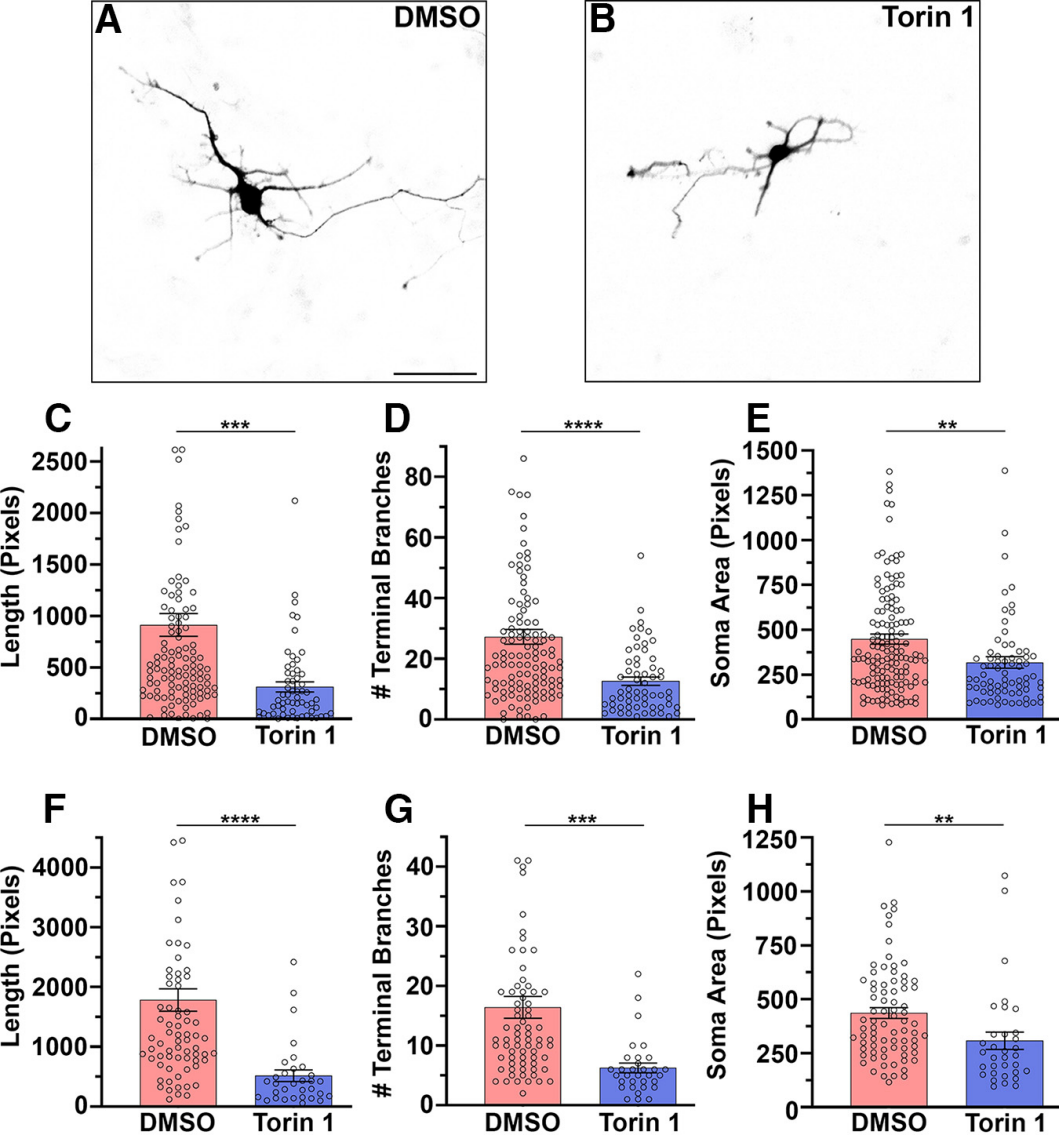
Torin 1-induced morphologic changes in Rheb^Y35L^ neurons. ***A***, ***B***, DIV3 tdTomato-positive Rheb^Y35L^ cortical neurons treated with 100 nm Torin 1 or vehicle. ***C***, ***D***, Automated measurement of total neurite length per cell (***C***) and terminal branch number per cell (***D***) using CellProfiler (DMSO: *n* = 116, Torin 1: *n* = 60). ***E***, Automated measurement of soma size using CellProfiler (DMSO: *n* = 144, Torin 1: *n* = 73). ***F***, ***G***, Manual measurement of total neurite length per cell (***F***) and terminal branch number per cell (***G***) using FIJI (DMSO: *n* = 74, Torin 1: *n* = 32). ***H***, Manual measurement of soma size using FIJI (soma size; DMSO: *n* = 78, Torin 1: *n* = 33). One to eight individual points were omitted from the DMSO condition by *y*-axis truncation to better visualize the mean (bar) and the change between conditions. Scale bar = 50 μm. 1 μm = 1.5384 pixels. DIV = days *in vitro*. **p < 0.01, ***p < 0.001, ****p < 0.0001. Significant differences were determined by an unpaired two-tailed Student’s *t* test.

Because of the high number of cells analyzed and the automated nature of the measurements, the comparison between groups (particularly involving DIV9 neurons, which had very extensive neurite arbors) are more easily visualized if the *y*-axis is truncated to omit one to eight upper limit cells from the graph. These were not omitted from analysis or statistics, and only represent a small fraction of the 375 (Fig. 2) or 176 (Fig. 3) cells analyzed. Number of visually omitted individual points per graph for Figure 2 by *y*-axis truncation: (DIV3 = 0 for all graphs), Figure 2*D*, DIV6 = 1, DIV9 = 7; Figure 2*E*, DIV6 = 2, DIV9 = 6; Figure 2*F*, DIV6 = 0, DIV9 = 3; Figure 2*G*, DIV6 = 0, DIV9 = 6. Number of visually omitted individual points per graph for Figure 3 by *y*-axis truncation: (Torin 1 = 0 for all graphs), Figure 3*C*, DMSO = 8; Figure 3*D*, DMSO = 4; Figure 3*E*, DMSO = 2; Figure 3*F*, DMSO = 6; Figure 3*G*, DMSO = 3; Figure 3*H*, DMSO = 0.

### Manual neurite and soma measurements

Images were uploaded to FIJI (ImageJ 1.53q) and neurites were traced using the Simple Neurite Tracer plug-in. Total length and number of terminal branchpoints per cell were extracted from FIJI based on the manual tracing. The freehand tracing tool in FIJI allowed for quantification of cell size by tracing the soma of tdTomato+ neurons and extracting the area.

### Statistical analysis

Statistical analyses using unpaired two-tailed Student’s *t* test and ordinary one-way ANOVA with Tukey’s *post hoc* test were performed using GraphPad Prism version 9.3.1.

## Results

Figure 1*A* illustrates the experimental paradigm from neuron labeling to analysis that constitute our new phenotypic assay. We performed IUE of one plasmid encoding both a constitutively active Y35L mutant Rheb and tdTomato into the lateral ventricle of E15 mouse embryos. We targeted E15 neural stem cells (radial glia) of the somatosensory cortex (SSC) that generate Layer II/III pyramidal neurons (Molyneaux et al., 2007; Lin et al., 2016; Sokolov et al., 2018). Rheb^Y35L^ is known to increase mTOR activity, resulting in neuron dysmorphogenesis including increased soma size and dendrite overgrowth (Zhao et al., 2019). Four days later, the SSC containing tdTomato-positive neurons were microdissected at postnatal day 0–1 and cultured onto a glass bottom 96-well plate at a density of 30,000 cells per well. This approach led to sparse tdTomato-positive neuron plating (Fig. 2*A–C*). On the indicated days *in vitro* (DIV), neurons were fixed, and immunocytochemistry was performed for tdTomato to amplify neurite labeling. Next, neurons were imaged in an automated fashion using an IN Cell Analyzer 2200 Imaging System. Following image acquisition, morphologic measurements were performed by feeding the IN Cell Analyzer 2200 images into a pipeline of customized modules in CellProfiler. These modules include various postprocessing steps such as identifying structures and adaptive thresholding (detailed in Materials and Methods; Fig. 1*B–E*). To validate this phenotypic assay, we examined neurite outgrowth over time and tested the impact of the mTOR blocker, Torin 1, on neurite outgrowth and soma size in a hyperactive mTOR condition. This experimental paradigm was completed in under two weeks including analysis.

### Automatic versus manual assessment of neurite outgrowth *in vitro* over time

To measure baseline morphologic changes in Rheb^Y35L^ neurons over time, we compared neurite outgrowth at DIV3, DIV6, and DIV9. Using the protocol outlined in Figure 1, significant increases in neurite length and complexity were identified over time. Total neurite length per cell increased by 99% from DIV3 to DIV6 (*p* < 0.01) and 74% from DIV6 to DIV9 (*p* < 0.0001; *N* = 3 wells for each time point for all experiments; Fig. 2*A–D*). Additionally, the number of terminal branches per cell, an indicator of neurite complexity, increased by 79% from DIV3 to DIV6 (*p* < 0.05) and 93% from DIV6 to DIV9 (*p* < 0.0001; Fig. 2*E*). To validate our automated analysis, we manually traced and measured the neurites of labeled neurons using FIJI software. This confirmed the changes in neurite morphology found using the automated pipeline, with an increase in the total neurite length per cell (DIV3-to-DIV6: 127%, *p* < 0.001; and DIV6-to-DIV9: 79%, *p* < 0.0001) and the number of terminal branches per cell (DIV3-to-DIV6: 57%, *p* < 0.05; and DIV6-to-DIV9: 71%, *p* < 0.0001; Fig. 2*F*,*G*). These data suggest that the automated analysis accurately recapitulates manual analysis and is sensitive and robust enough to detect statistically significant differences in neurite length and complexity over time.

### Automatic and manual assessment of Torin 1-induced morphologic changes in Rheb^Y35L^ neurons

Considering mTOR’s well-documented role in dendrite outgrowth, we chose the mTOR inhibitor Torin 1 to assess the efficacy of using our pipeline to monitor drug-induced morphologic changes. We repeated the protocol outlined in Figure 1 and treated cultured Rheb^Y35L^ neurons daily with 100 nm Torin 1 or vehicle until DIV3 (equimolar DMSO, 0.01%). We chose to treat for 3 d considering neurites are not overly complex at this time point, allowing less neurite overlap and more accurate automated detection. We found that Torin 1 reduced total neurite length per cell by 66% (*p* < 0.001; Fig. 3*A–C*). Torin 1 also decreased the number of terminal branches per cell by 54% (*p* < 0.0001; Fig. 3*D*). As mTOR activity influences cell size, we used a second modified CellProfiler pipeline to automatically measure soma size and found a 29% reduction following Torin 1 treatment (*p* < 0.01; Fig. 3*E*). To confirm that these changes were accurately measured, we manually traced and measured neurites using FIJI software. The manual analysis reflected the results of the automated analysis, showing Torin 1 reduced the total neurite length per cell (71% decrease; *p* < 0.0001), the number of terminal branches per cell (62% decrease; *p* < 0.001), and soma size (30% decrease; *p* < 0.01; Fig. 3*F–H*). Thus, our automated phenotypic assay is a viable alternative to manual analysis to efficiently analyze the morphologic effect of drugs on sparsely labeled neurons.

## Discussion

Here, we describe a versatile novel medium-throughput assay to investigate morphologic changes in primary mouse cortical neurons in an automated fashion. As opposed to cell lines, primary neuronal cultures offer a more clinically relevant model for studying neurologic disorders. We also used IUE, which allows us to express a plasmid of our choice that encodes constitutively active Rheb to increase mTOR activity, as reported in several neurodevelopmental disorders, and assess the impact of drug treatment on neuron morphology.

We validated our experimental paradigm by successfully measuring and quantifying changes in neurite outgrowth over time and following Torin 1 treatment using the CellProfiler pipeline. As expected, our assay detected increased neurite length and complexity over time and Torin 1 reduced neurite length, complexity, and soma size compared with vehicle treated neurons. To further confirm the accuracy of the automated measurements, we performed traditional manual tracings using FIJI Simple Neurite Tracer (Schindelin et al., 2012). The manual analysis gave similar results to those with the automated analysis. However, while manual tracing took over a week, our automated approach took hours. It is nevertheless important to note the discrepancy in mean values recorded using the automated versus the manual method. The automated method underestimated the total length and complexity compared with the manual tracing method. This discrepancy is because of the thresholding applied using automated analysis. A threshold is applied to avoid false neurite labeling (e.g., because of neurite crossing), resulting in the omission of fainter distal portions of the neurites. However, the changes between experimental conditions remain consistent with automated and manual analyses. Collectively, our automated paradigm is a viable alternative to the traditional manual analysis for quantifying neuron morphology in a more efficient manner.

To label cells for automated neurite imaging and tracing, we used IUE. Other labeling techniques can be combined with our approach such as sparse transfection, nucleofection, lentiviral infection of cultured rodent neurons or human iPSC-derived neurons, and AAV infection *in vivo*. It can also be combined with the use of transgenic mice or rats. All these techniques allow sparse labeling and the targeting of neurons or glia instead of using cell lines differentiated into a neuron-like state that do not express all neuronal or glial properties (Dravid et al., 2021; Schikora et al., 2021). Drug screening may thus be more clinically translatable. The major advantage of IUE over plasmid transfection, nucleofection, and lentivirus infection *in vitro,* is the specificity of the labeled cell type and region targeted without the need for using specific promoters. Here, we targeted E15 cortical radial glia that generate Layer II/III pyramidal neurons (Molyneaux et al., 2007; Lin et al., 2016; Sokolov et al., 2018). IUE at an earlier time point would allow targeting of deep layer pyramidal neurons, and IUE at later timepoints (E18) would preferentially label astrocytes (Molyneaux et al., 2007). Targeting the ganglionic eminence would label interneurons (Anderson et al., 1997; Kepecs and Fishell, 2014). Another advantage of IUE over the above mentioned *in vitro* labeling approaches is that cell viability is increased considering IUE occurs days before culturing the neurons (Blackmore et al., 2010; Sharma et al., 2012). Compared with transgenic mice, IUE is simply more versatile in expressing plasmids that encode any protein of interest or knock-down systems (shRNA or CRISPR/Cas9) without the need to generate double or triple transgenic mice. Finally, IUE is comparable to *in vivo* AAV considering that the cost of AAV production has decreased. Specific promoters can allow AAV to be expressed in excitatory versus inhibitory neurons (interneurons) versus different types of glia. The promoters may not be yet specific enough to achieve labeling of Layer II/III versus IV or V pyramidal neurons, but most screens may not need this level of specificity.

Our assay, however, has a couple of minor limitations. The CellProfiler algorithm does not efficiently differentiate between axons and dendrites. This did not affect data from our studies since mTOR affects both axon and dendrite growth (Kumar et al., 2005; Gong et al., 2015). For future studies examining the effect of drugs on either axon or dendrite growth, it would be important to perform immunocytochemistry for specific neurite markers, such as MAP2 for dendrites and tau for axons, and optimize the CellProfiler modules to only measure MAP2 or tau positive objects. Another limitation is that IUE requires technical expertise in handling and manipulating embryonic tissues as well as precise injection capability. A level of consistency with the IUE and microdissection procedure is essential for optimally labeling cultured neurons.

In conclusion, we have developed a new phenotypic assay that can screen the morphologic effects of compounds on specific neuronal populations in a robust and selective fashion *in vitro*. The newly designed pipelines in CellProfiler can be adapted to analyze the morphology of different populations of neurons and glia as well as non-neuronal cells *in vitro*. Finally, the assay is medium-throughput and can provide clinically relevant data on ∼32 compounds in under two weeks.

## Synthesis

Reviewing Editor: Jeffery Twiss, University of South Carolina Decisions are customarily a result of the Reviewing Editor and the peer reviewers coming together and discussing their recommendations until a consensus is reached. When revisions are invited, a fact-based synthesis statement explaining their decision and outlining what is needed to prepare a revision will be listed below. The following reviewer(s) agreed to reveal their identity: Erna van Niekerk.

This manuscript combines several validated techniques to create a new pipeline for in vivo viral vector delivery, in combination with cell culture manipulation(s) such as drug screening in vitro, where the output measure is changes in morphology using automated software. The appeal of this medium-throughput methodological pipeline is that it is provides assessment for effects of potential disease-modifying compounds/manipulations on neuronal morphology. The authors aim to demonstrate that this pipeline robustly replicates manual imaging and morphological assessments. Although the pipeline was only tested in one population of neurons, theoretically, this could be applied to a myriad of neurons involved in different diseases where identifying drugs or small molecules may provide a rapid path towards clinical translation. So both the reviewers and reviewing editor feld that your approach could significantly increase the throughput for drug screening, and thus provide a tool to increase the discovery of clinically relevant compounds for neurological disorder treatments. However as a new methods report, the work is limited by several points. The authors heavily rely on using automated software in this manuscript, but they did not include detailed methods on how this was achieved - specifically, there are no methods describing two critical components for your approach - the high content imaging and the use of Cell Profiler. One reviewer noted that you combine in utero electroporation with cell culture techniques to identify phenotypic changes in neurons following treatment of either drugs or viral vectors, but did not take the step to combine these techniques into one readout. I agree that would strengthen your manuscript, but do not feel that is necessary to address for resubmission - perhaps that could be included in the discussion for future directions.

Major Points from the reviewers:

1. Lines 40-41 of the abstract state that both automated and manual analyses showed increased neurite length over time and decreased neurite growth with Torin-1. However, only automated neurite length measurements are shown in the Figures (Figures 2D and 3C). Manual measurements of neurite length were not conducted in this study. A manual analysis of neurite length needs to be completed and graphed.

2. The Materials and Methods do not describe the microscope or imaging parameters used. Details should be added about the high content imager employed and the parameters used for acquisition (e.g., objective/magnification, exposure time, etc). Further, it is unclear if image acquisition was done automated as well, or through using a regular microscope. Please provide method details on how imaging acquisition was accomplished.

3. An Automated Neurite tracing section needs to be added to the methods section. With automated software packages, some level of manual verification and correction is often necessary to ensure accuracy of the traced neurons. For example, cell body diameter cutoffs need to be specified, along with background noise, and what is considered a neurite. The authors describe using inCell Analyzer 2200 for automated tracing in figure 2, and then later switch to another software package Cell Profiler for figure 3. The rationale for using two different software packages needs to be clarified, along with methods for both software settings.

4. The Materials and Methods do not describe the “pipeline of customized modules on CellProfiler” used for the analysis (line 188). Since this is a Methods paper, details should be included on these “customized” steps employed in CellProfiler. For example, information on how the post-processing steps (e.g., neurite object, threshold, skeletonized) were customized and the specific analyses performed (e.g., number of terminal branches) should be included. This is needed to evaluate the methodology and allow future investigators to replicate this pipeline.

5. Figures 2 and 3. CellProfiler was used to determine the neurite length in pixels and the number of terminal branches (Figures 2D and 2E, 3C and 3D). The manual validation of these parameters is through a Sholl analysis (Figures 2F and 3F), and the Methods section states that this is the number of total intersections per cell (at 10um intervals). However, it is unclear how a Sholl analysis validates CellProfiler’s analysis of the number of terminal branches. The Sholl analysis counts the number of times neurites intersect 10um concentric circles; thus, with a Sholl analysis, one long neurite (with no branches) would be counted multiple times if it crosses multiple circles. However, the CellProfiler analysis (number of terminal branches) would count that same neurite as “1” because it only has one terminal branch. Although both types of analyses measure complexity, they measure it differently. Sholl analysis also does not directly measure neurite length. Thus, it would improve the paper to also manually conduct the exact same analyses done in CellProfiler (i.e., neurite length and number of terminal branches) and include these graphs as well. This would increase the validity of the novel customized measurements in CellProfiler the authors have developed.

6. Figure 2. The legend states that the bar graphs are truncated for the DIV 6 and DIV 9 time points “to better visualize the progressive change overtime.” More details are needed on this truncation here or in the Methods section. Alternatively, the authors could make these graphs with a discontinuous y-axis (and compress the currently truncated points), which would allow all data points to be shown and retain this better visualization of the change over time.

7. Figure 2a-c, it is unclear what the images were labeled with. Please specify if these images represent tdTomato. Figure 2d, is length the length per cell reported? If so, please specify length as total length per cell. Figure 2e, please specify how number of terminal branches were identified. If through inCell Analyzer, how was that achieved.

8. In Figure 3 a,b no IUE was used. For these images, it is unclear what label was used to identify neurons, please specify. Figure 3c, is length the length per cell reported? If so, please specify length as total length per cell. Figure 3d, please specify how number of terminal branches were identified. If through inCell Analyzer, how was that achieved.

9. Statistics appear to be correct from the general Methods section. However, the specific statistical tests used in Figures 2 and 3 should be added to the legends. This will allow the reader to determine if the appropriate statistical test was used for each analysis.

10. Neuron plating density also needs to be provided. For this method to be adopted by other groups, plating density can be profoundly affected by automated tracing software and accuracy in predicting neurite length becomes challenging in high density cultures.

11. The authors describe culturing pyramidal neurons from layer II/II, however do not provide glutamatergic evidence since these layers of the cortex contains both interneurons and pyramidal cells.

Minor Comments from Reviewers:

1. Lines 33, 38, 40, 168, 190, 191 - please change “dendrite” to “neurite” because all neurites, not just dendrites, were analyzed in the paper.

2. Line 142: It is more translational if the centrifugation speed is reported in g force, rather than RPM.

3. Please add the number of separate timed pregnant animals that underwent IUE.

4. IUE is perhaps overemphasized in the Discussion. In fact, the rest of this pipeline is powerful. It can be used with other methodologies under certain conditions, such as cultured neurons from transgenic animals or sparsely transfected/transduced neurons. Although it is important to keep in the Discussion why IUE has benefits, adding that the rest of the pipeline can be used with other techniques would strengthten the paper.

5. Figure 2 - the title and description of this figure would be improved by replacing the word “diseased” with “mTOR upregulation” or “Overexpression of RhebY35L“

6. CellProfiler provides an output in pixels (not micrometers), but the manual measurements were calculated in micrometers (for example, see Figures 3E and 3G). The comparison will be improved if the automated and manual measurements are in the same units. The microscope has a pixel-to-micrometer conversion factor so that pixels can be converted into micrometer values manually after the CellProfiler output in pixels. Alternatively, the manual measurements could be conducted without a conversion factor, and thus would be in pixels.

7. Line 224. Based on the first part of this sentence and the figure legend, I believe that “neurite outgrowth” should be “number of crossings.”

## Author Response

We thank the reviewers for their support and appreciation of our study and for providing a very valuable set of suggestions and comments. We have addressed all the comments in the revised manuscript. The specific responses are outlined point-by-point below and changes are highlighted in blue in the revised manuscript. A clean version of the manuscript is also provided.

Synthesis Statement for Author:

This manuscript combines several validated techniques to create a new pipeline for in vivo viral vector delivery, in combination with cell culture manipulation(s) such as drug screening in vitro, where the output measure is changes in morphology using automated software. The appeal of this medium-throughput methodological pipeline is that it is provides assessment for effects of potential disease-modifying compounds/manipulations on neuronal morphology. The authors aim to demonstrate that this pipeline robustly replicates manual imaging and morphological assessments. Although the pipeline was only tested in one population of neurons, theoretically, this could be applied to a myriad of neurons involved in different diseases where identifying drugs or small molecules may provide a rapid path towards clinical translation. So both the reviewers and reviewing editor felt that your approach could significantly increase the throughput for drug screening, and thus provide a tool to increase the discovery of clinically relevant compounds for neurological disorder treatments. However as a new methods report, the work is limited by several points. The authors heavily rely on using automated software in this manuscript, but they did not include detailed methods on how this was achieved - specifically, there are no methods describing two critical components for your approach - the high content imaging and the use of Cell Profiler. One reviewer noted that you combine in utero electroporation with cell culture techniques to identify phenotypic changes in neurons following treatment of either drugs or viral vectors, but did not take the step to combine these techniques into one readout. I agree that would strengthen your manuscript, but do not feel that is necessary to address for resubmission - perhaps that could be included in the discussion for future directions.

Response: Thank you for the positive comments and the suggestions. As detailed in the specific comments, we provide a better description of the techniques and analysis methods. We also modified the discussion to address one of the reviewers’ comments regarding the viral vectors that stems from a lack of clarity.

Major Points from the reviewers:

Comment 1. Lines 40-41 of the abstract state that both automated and manual analyses showed increased neurite length over time and decreased neurite growth with Torin-1. However, only automated neurite length measurements are shown in the Figures (Figures 2D and 3C). Manual measurements of neurite length were not conducted in this study. A manual analysis of neurite length needs to be completed and graphed.

Response: As suggested, we added a graph of neurite length acquired using manual analysis.

Comment 2. The Materials and Methods do not describe the microscope or imaging parameters used. Details should be added about the high content imager employed and the parameters used for acquisition (e.g., objective/magnification, exposure time, etc). Further, it is unclear if image acquisition was done automated as well, or through using a regular microscope. Please provide method details on how imaging acquisition was accomplished.

Response: Thank you for catching the lack of details about imaging. Imaging acquisition was done in an automated fashion using an InCell Analyzer 2200. We added a section entitled “Automated Imaging” in the methods. We provided details of the microscope used and the imaging parameters including the filters, the objective, and exposure time.

Comment 3. An Automated Neurite tracing section needs to be added to the methods section. With automated software packages, some level of manual verification and correction is often necessary to ensure accuracy of the traced neurons. For example, cell body diameter cutoffs need to be specified, along with background noise, and what is considered a neurite. The authors describe using inCell Analyzer 2200 for automated tracing in figure 2, and then later switch to another software package Cell Profiler for figure 3. The rationale for using two different software packages needs to be clarified, along with methods for both software settings.

Response: As suggested we added a Method section about the automated neurite tracing with details on parameters. We have contacted the operators of the CellProfiler website, and they agreed to adding our pipelines used in this paper once we can provide a citation for this paper. If accepted, we will send the pipelines at once, that way they can be downloaded for use. We also added the sentence “Pipeline files can be found on the CellProfiler website for download” to the methods. We apologize for the confusion in the figures. InCell Analyzer 2000 is the imaging microscope and CellProfiler is the software package that is now described in the method section (see point #2). CellProfiler is for the automated neurite and soma analyses. By adding descriptions in the method, this should help prevent confusion.

Comment 4. The Materials and Methods do not describe the “pipeline of customized modules on CellProfiler” used for the analysis (line 188). Since this is a Methods paper, details should be included on these “customized” steps employed in CellProfiler. For example, information on how the post-processing steps (e.g., neurite object, threshold, skeletonized) were customized and the specific analyses performed (e.g., number of terminal branches) should be included. This is needed to evaluate the methodology and allow future investigators to replicate this pipeline.

Response: We fully agree with the reviewer. This has been addressed (also suggested in Comment 3 above).

Comment 5. Figures 2 and 3. CellProfiler was used to determine the neurite length in pixels and the number of terminal branches (Figures 2D and 2E, 3C and 3D). The manual validation of these parameters is through a Sholl analysis (Figures 2F and 3F), and the Methods section states that this is the number of total intersections per cell (at 10um intervals). However, it is unclear how a Sholl analysis validates CellProfiler’s analysis of the number of terminal branches. The Sholl analysis counts the number of times neurites intersect 10um concentric circles; thus, with a Sholl analysis, one long neurite (with no branches) would be counted multiple times if it crosses multiple circles. However, the CellProfiler analysis (number of terminal branches) would count that same neurite as “1” because it only has one terminal branch. Although both types of analyses measure complexity, they measure it differently. Sholl analysis also does not directly measure neurite length. Thus, it would improve the paper to also manually conduct the exact same analyses done in CellProfiler (i.e., neurite length and number of terminal branches) and include these graphs as well. This would increase the validity of the novel customized measurements in CellProfiler the authors have developed.

Response: The reviewer has a valid point. We thus manually quantified total neurite length per cell and terminal branch number using the manual tracings. These data are now presented in Figure 2 and 3. As such, we removed the number of total intersections per cell originally obtained with the Sholl analysis from Figure 2 and 3 (replaced by the number of terminal branches).

Comment 6. Figure 2. The legend states that the bar graphs are truncated for the DIV 6 and DIV 9 time points “to better visualize the progressive change overtime.” More details are needed on this truncation here or in the Methods section. Alternatively, the authors could make these graphs with a discontinuous y-axis (and compress the currently truncated points), which would allow all data points to be shown and retain this better visualization of the change over time.

Response: To address this comment, we extended the y axis of our truncated graphs so that only a small fraction (1-8 individual points) are omitted out of 395 (Figure 2) or 176 (Figure 3) points, and in the methods under automated measurements, we have listed the number of points removed by condition for each individual graph. This is just a visual omission to allow the reader to easily see the mean value for all conditions based on the bar size and the change between conditions. No points are omitted from statistics or reported values. We considered adding a discontinuous y-axis, but we would lose additional datapoints as there are no large gaps.

Comment 7. Figure 2a-c, it is unclear what the images were labeled with. Please specify if these images represent tdTomato. Figure 2d, is length the length per cell reported? If so, please specify length as total length per cell. Figure 2e, please specify how number of terminal branches were identified. If through inCell Analyzer, how was that achieved.

Response: Thank you for pointing out the lack of clarity. The images indeed represent tdTomato. The total neurite length is indeed per cell. We added details in the methods about the analysis using Cell Profiler (InCell Analyzer is the microscope and software acquisition package). We made the required changes in the figure legends and results to provide more clarity.

Comment 8. In Figure 3 a,b no IUE was used. For these images, it is unclear what label was used to identify neurons, please specify. Figure 3c, is length the length per cell reported? If so, please specify length as total length per cell. Figure 3d, please specify how number of terminal branches were identified. If through inCell Analyzer, how was that achieved.

Response: We apologize for the confusion. IUE was used for Figure 3 in the same manner as Figure 2, and the RhebY35L neurons are labeled with tdTomato. We updated the wording in the figure legend and results to better convey that the methods (experimental protocol, imaging, and analysis) were the same as in Figure 2. Both Figure 2 and 3 use the same protocol outlined in Figure 1.

Comment 9. Statistics appear to be correct from the general Methods section. However, the specific statistical tests used in Figures 2 and 3 should be added to the legends. This will allow the reader to determine if the appropriate statistical test was used for each analysis.

Response: As suggested, we added the statistical method used in the figure legends.

Comment 10. Neuron plating density also needs to be provided. For this method to be adopted by other groups, plating density can be profoundly affected by automated tracing software and accuracy in predicting neurite length becomes challenging in high density cultures.

Response: We apologize for not highlighting the neuron plating density, we completely agree that this is very important to report due to the sensitivity of the automated measurements. We have added this information to the results (30,000 cells per well), in addition to it being in the methods section (line 149 of original submission).

Comment 11. The authors describe culturing pyramidal neurons from layer II/II, however do not provide glutamatergic evidence since these layers of the cortex contains both interneurons and pyramidal cells.

Response: This comment stems from a lack of clarity while describing the method and in utero electroporation (IUE). IUE was used for all the experiments. We performed IUE at embryonic day 15 and send the plasmids toward the cortical ventricle zone. As reported by many labs, including ours, this selectively targets and express plasmids in cortical radial glia that generate layer II/III pyramidal neurons. Interneurons that are generated in the ganglionic eminence are not labelled. We provided several publications following the sentence in the results: “We targeted E15 cortical neural stem cells (e.g., cortical radial glia) of the somatosensory cortex (SSC) that generate layer II/III pyramidal neurons”. Added references:

Molyneaux, B. J., Arlotta, P., Menezes, J. R., & Macklis, J. D. (2007). Neuronal subtype specification in the cerebral cortex. Nat Rev Neurosci, 8(6), 427-437. https://doi.org/10.1038/nrn2151

Sokolov, A. M., Seluzicki, C. M., Morton, M. C., & Feliciano, D. M. (2018). Dendrite growth and the effect of ectopic Rheb expression on cortical neurons. Neurosci Lett, 671, 140-147. https://doi.org/10.1016/j.neulet.2018.02.021

Lin, T. V., Hsieh, L., Kimura, T., Malone, T. J., & Bordey, A. (2016). Normalizing translation through 4E-BP prevents mTOR-driven cortical mislamination and ameliorates aberrant neuron integration. Proc Natl Acad Sci U S A, 113(40), 11330-11335. https://doi.org/10.1073/pnas.1605740113

Minor Comments from Reviewers:

Comment 1. Lines 33, 38, 40, 168, 190, 191 - please change “dendrite” to “neurite” because all neurites, not just dendrites, were analyzed in the paper.

Response: As suggested, we changed.

Comment 2. Line 142: It is more translational if the centrifugation speed is reported in g force, rather than RPM.

Response: This is changed as suggested.

Comment 3. Please add the number of separate timed pregnant animals that underwent IUE.

Response: This has been added.

Comment 4. IUE is perhaps overemphasized in the Discussion. In fact, the rest of this pipeline is powerful. It can be used with other methodologies under certain conditions, such as cultured neurons from transgenic animals or sparsely transfected/transduced neurons. Although it is important to keep in the Discussion why IUE has benefits, adding that the rest of the pipeline can be used with other techniques would strengthen the paper.

Response: We agree with the reviewer that our imaging and analysis approaches can be easily combined with other labeling techniques. The discussion was adjusted accordingly by de-emphasizing IUE and mentioning other labeling techniques.

Comment 5. Figure 2 - the title and description of this figure would be improved by replacing the word “diseased” with “mTOR upregulation” or “Overexpression of RhebY35L“

Response: We replaced the word diseased with RhebY35L-overexpressing pyramidal neurons.

Comment 6. CellProfiler provides an output in pixels (not micrometers), but the manual measurements were calculated in micrometers (for example, see Figures 3E and 3G). The comparison will be improved if the automated and manual measurements are in the same units. The microscope has a pixel-to-micrometer conversion factor so that pixels can be converted into micrometer values manually after the CellProfiler output in pixels. Alternatively, the manual measurements could be conducted without a conversion factor, and thus would be in pixels.

Response: This is a good point. We now provide all the measurements in pixels and provide the size of pixels (in μm) in the figure legend.

Comment 7. Line 224. Based on the first part of this sentence and the figure legend, I believe that “neurite outgrowth” should be “number of crossings.”

Response: Thank you. We changed as suggested.
